# Anti-Inflammatory Activity of *Artemisia vulgaris* Leaves, Originating from Three Different Altitudes of Nepal

**DOI:** 10.1155/2021/6678059

**Published:** 2021-06-22

**Authors:** Jitendra Pandey, Sushma Bhusal, Laxman Nepali, Maya Khatri, Rasmita Ramdam, Himal Barakoti, Paras Mani Giri, Dhakaraj Pant, Pramod Aryal, Rabindra Kumar Rokaya, Ravin Bhandari

**Affiliations:** ^1^Department of Pharmacy, Crimson College of Technology, Affiliated to Pokhara University, Butwal, Rupandehi 32900, Nepal; ^2^Department of Pharmacy, Purbanchal University, Biratnagar 56613, Nepal; ^3^Siddhartha Pharmaceutical Private Limited, Rupandehi 32900, Nepal; ^4^Department of Medical Laboratory Science, School of Health and Allied Sciences, Pokhara University, Pokhara 33370, Nepal; ^5^Department of Pharmacology, Karnali Academy of Health Science, Jumla, Chandannath 21200, Nepal

## Abstract

This study aimed to evaluate and compare the in vivo chronic anti-inflammatory efficacy, from the ethyl acetate and ethanolic extracts of *Artemisia vulgaris* leaves, grown at three different altitudes in Nepal, by formalin-induced paw edema in Swiss albino mice. Edema was induced on the mice paw by administering 0.2% of formalin injection. Indomethacin was used as a standard drug at the concentration of 5 mg/kg of body weight. Ethyl acetate and ethanolic leaves extract, at the concentration of 200 mg/kg and 400 mg/kg, were used as test drugs. Standard drug and all the extracts were administered 30 min before formalin injection. The paw thickness was measured at 0, 1, 2, 3, 24, 48, and 72 hours after formalin injection, using a Vernier caliper. It was observed that both ethyl acetate and ethanolic extract from all the altitudes exhibited significant inhibition of paw edema (*p* < 0.05) induced by formalin. Maximum activity was shown by 400 mg/kg of the plant leaf extract taken from the temperate zone, with 54.05% of paw edema inhibition, and it is almost similar to the inhibition of standard drug (56.75%). Moreover, the ethanolic extract was found to be more effective than ethyl acetate extract in all the plant samples. The results suggested that the anti-inflammatory effect of *A. vulgaris* leaves increases with an increase in altitudes and this plant can be used as a useful source of medicine to treat chronic inflammation.

## 1. Introduction

Inflammation is a localized protective response of mammalians cells/tissues towards allergic or chemical irritation, injury, and infection. Development of inflammation is associated with an increased level of various endogenous biological molecules, including nitric oxide (NO), reactive oxygen species (ROS), prostaglandin E2 (PGE_2_), and cytokines [1]. Depending upon the type of stimulus and efficacy of the inflammatory reaction in removing damaged tissue and harmful stimuli, inflammation may be acute or chronic in nature [2]. The primary defensive mechanism of macrophages during inflammation is characterized by the release of antimicrobial agents and cellular signaling molecules. However, the inflated release of cellular mediators through macrophages can lead to host cell damage, resulting in different diseases like rheumatism, encephalitis, pneumonia, esophagitis, cancer, heart problems, and fibrosis [3–6]. Currently, either narcotic such as opioid analgesic or nonsteroidal anti-inflammatory drugs like corticosteroids, salicylates have been used for the management of pain and inflammatory conditions. However, they exhibit serious adverse and toxic effects. Moreover, the overall cost for the development of a synthetic novel drug is very high. Therefore, screening of potential anti-inflammatory drugs having fewer adverse effects on natural plant sources is an alternative way to overcome this problem [5].

*Artemisia vulgaris* L., also known as mugwort, is a perennial long-stemmed shrub belonging to the family Compositae. It comprises hardy herbs and shrubs known for their volatile oils. It is native to temperate Europe, Asia, and northern Africa, but is also present in North America as an invasive weed [7]. In Ayurveda, *A. vulgaris* is a source of the snakebite antidote drug “nagdaun.” Traditionally aerial parts of *A. vulgaris* have been extensively used as anthelmintic, antiseptic, antispasmodic, antidiabetic, antiepileptic, vermicides, antiepilepsy, and antidepressant [8, 9]. The root has different uses, as a tonic for psychoneuroses, depression, autonomic neurosis, irritability, restlessness, insomnia, and anxiety. In China, *A. vulgaris* is used mostly for moxibustion. *A. vulgaris* is believed to stimulate blood flow and Qi at specific points on the skin, sometimes acupuncture points [10]. Different parts of *A. vulgaris* were reported to have broad biological activities such as antimicrobial, antihypertensive, antispasmodic and bronchodilator, hepatoprotective, antidepressant, xanthine oxidase inhibitor, and antioxidant [11]. The study on the regional variation of anti-inflammatory activity on this plant has not been conducted yet. Hence, this study aims to perform a comparative study of the in vivo anti-inflammatory activity of *A. vulgaris* obtained from three different geographical areas.

## 2. Materials and Methods

### 2.1. Chemicals Required

Indomethacin was purchased from S.R. Laboratories Pvt. Ltd., Kathmandu. Formaldehyde (Thermo Fisher scientific, India Pvt., Ltd., Mumbai), ethyl acetate, ethanol (Hangshu Kangyvan Chemical, China), and water were prepared in laboratory with distilled water plant.

### 2.2. Instruments

They are autoclave (S. M. Scientific Instruments (P) Ltd., Delhi), rotary evaporator (R-210/215, BUCHI Labortechnik AG, Switzerland), digital balance (ATX224, SHIMADZU Corporation, Philippines), grinder and distilled Water (DW) plant, hot air oven (S. M. Scientific Instruments (P) Ltd., Delhi), incubator (S. M. Scientific Instruments (P) Ltd.), refrigerator (GL-M492YLG), and sonicator (INDOSATI Scientific Instruments (P) Ltd., Delhi).

### 2.3. Plant Materials

The plant parts were collected from three different regions of Nepal, namely, Tamnagar, Rupandehi (tropical region, 350 m above sea level), Dhanchaur Arghakhanchi (subtropical region, 1829 m above the sea level), and Khalanga, Jumla (temperate region, 2518 m above sea level). Plant materials were taxonomically identified by Mr. Hom Nath Pathak, Prithivi Narayan Campus, Tribhuvan University, Nepal (identification letter number (FR-2015/26)) together with a literature comparison. The voucher specimen of the identified plants has been preserved in Crimson College of Technology, Pharmacognosy Lab (voucher specimen number: CCT-HRB-2018/169). Detailed information about the collection of plant material is given in Table 1.

### 2.4. Preparation of Plant Extract

The plant parts were collected, cleaned, and converted to a fine powder after proper shade drying. A double cold maceration extraction procedure was performed by taking 200 g leaves of different regions, soaked with 1000 mL of ethyl acetate and ethanol in different conical flasks with occasional shaking for 72 hours. Liquids were strained and filtered. The process was repeated up to double maceration and the filtrate was mixed and then dried by using a rotary vacuum evaporator at a temperature of 40°C. The extract was placed in glass vials and extractive yields were determined. All the airtight vials were preserved in the refrigerator at 4°C until use.

### 2.5. Animals Used and Ethical Approval

To conduct animal experiments, Swiss albino mice of either sex weighing 25–35 g were used. All the mice were continuously provided with standard feed and water ad libitum. They were accommodated inside clean polypropylene maintaining uniform room temperature (22 ± 1°C), with a regular 12/12 h dark/light cycle until the experiment is completed. Before the experimental analysis, all the animals were acclimatized to laboratory conditions. Experimental procedures, animal handling, and care were conducted according to the official ethical guidelines of Nepal [12–14]. All experimental mice were given 35% CO_2_ euthanasia after completing the analysis. The experimental protocol was authenticated by the Institutional Review Committee of Pokhara University (reference number: 130-089-076).

### 2.6. Acute Toxicity Studies

Acute toxicity in mice was investigated by adopting the Organization of Economic Cooperation and Development (OECD) guideline 425 [15]. A total of fifteen groups of Swiss Albino mice, each group containing five mice (*n* = 5), were formed. Among them, one was a normal control group and fourteen were test groups. The normal control group was supplied with normal saline and all test groups were provided with plant extract (500, 1000, and 2000 mg/kg, p.o.) at 10 mL/kg. Any toxic symptoms and mortality were monitored every 1 h for the next 6 h and total body weight was measured on days 1, 7, and 14 after treatment.

### 2.7. Anti-Inflammatory Activity Test

#### 2.7.1. Administration of Test Sample

The test extract was given as suspension, in 0.5% of tween 80 solutions [16].

#### 2.7.2. Preparation of Formalin Solution [16]

For this, 2 mL of formaldehyde was poured into a 100 mL volumetric flask and diluted up to exact 100 mL by using distilled water to prepare a 2% v/v fresh formalin solution.

#### 2.7.3. Formalin-Induced Edema in Mice Paw

Anti-inflammatory activity was evaluated by adopting the previously established formalin-induced paw edema method [7, 16–18]. Swiss albino mice were divided into 15 groups (5 animals in each group). Animals of all groups were injected with 0.2 mL of 2% v/v formalin, in the right hind paw, prepared by using distilled water.

  Group I animals (normal control) received 500 *μ*L of 0.5% tween 80 only.
  Group II animals (formalin control) received distilled water p.o., 30 min prior to formalin injection.
  Group III, the standard reference group, was given p.o. an aqueous solution of indomethacin (5 mg/kg), 30 min prior to formalin injection.
  Groups IV and V received p.o. ethanolic extract of *A. vulgaris* of the tropical region, 200 mg/kg and 400 mg/kg of 0.5% tween 80, respectively.
  Groups VI and VII received ethyl acetate extract of *A. vulgaris* of the tropical region, 200 mg/kg and 400 mg/kg of 0.5% tween 80, respectively.
  Groups VIII and IX received p.o. ethanolic extract of *A. vulgaris* of the subtropical region, 200 mg/kg and 400 mg/kg of 0.5% tween 80, respectively.
  Groups X and XI received p.o. ethyl acetate extract of *A.* vulgaris of the subtropical region, 200 mg/kg and 400 mg/kg of 0.5% tween 80, respectively.
  Groups XII and XIII received p.o. ethanolic extract of *A. vulgaris* of the temperate region, 200 mg/kg and 400 mg/kg of 0.5% tween 80, respectively.
  Groups XIV and XV received p.o. ethyl acetate extract of *A. vulgaris* of the temperate region, 200 mg/kg and 400 mg/kg of 0.5% tween 80, respectively.

All the extracts were administered 30 min prior to formalin injection. The treatment was continued for three consecutive days at a fixed time but formalin was injected only on the first day. The paw volume of the mice was measured by using a vernier caliper and observed before and 1 h, 2 h, 3 h, 24 h, 48 h, and 72 h after formalin injection. The percentage inhibition of edema was calculated as follows:

(1) $$\begin{array}{c} \text{\% inhibition}=\frac{\text{mean paw inflammation of control}-\text{mean paw inflammation of test }}{\text{mean paw inflammation of control}}\times 100. \end{array}$$

### 2.8. Statistical Analysis

The calculation of the average edema for the anti-inflammatory was based on the expression of numerical data as mean ± SD and was evaluated by two-tailed Student's *t*-test using Microsoft excel. The results obtained were compared with the control group. A *p* < 0.05 was considered statistically significant.

## 3. Results

### 3.1. Extractive Yield Value

The extractive yield of *A. vulgaris* leaves from different climatic zones in ethyl acetate and ethanol is given in Table 2.

### 3.2. Acute Toxicity Test

After the continuous monitoring of the experimental animal, no toxicity was observed. None of the groups reported mortality after different doses (0.2, 0.4, 0.8, and 1 g/kg) of plant extract ingestion. Symptoms of toxicity such as sitting at the corner and paw licking were precipitated only at a dose of 1000 mg/kg body weight.

### 3.3. Anti-Inflammatory Activity

**Figure 1:**
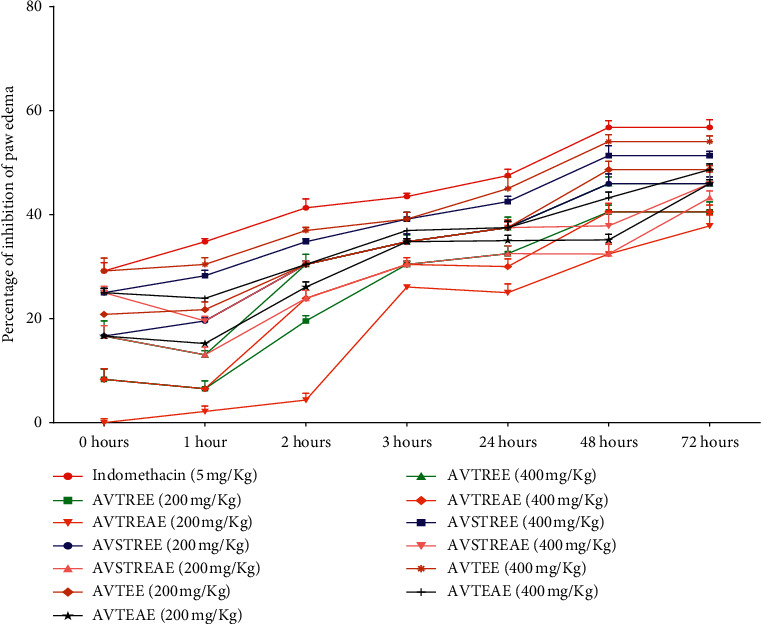
Graphical representations for percentage inhibition of mice paw volume by indomethacin, ethyl acetate, and ethanolic *A. vulgaris* extract from different altitudes on time course curve. Each point indicates mean ± SEM (*n* = 5); *p* < 0.05 in comparison to the formalin control group.

**Figure 2:**
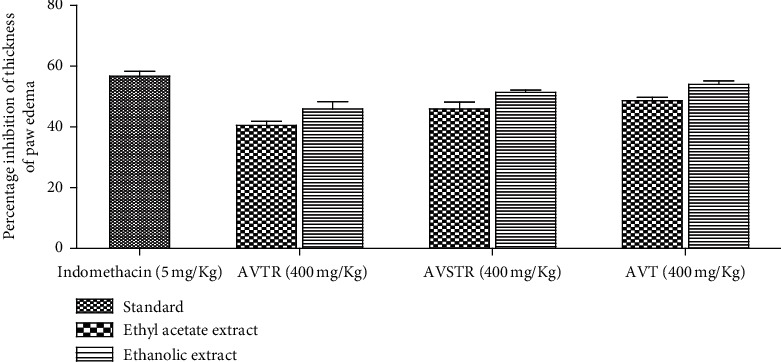
Comparison of paw edema volume inhibition among indomethacin, ethyl acetate, and ethanolic extract of *A. vulgaris* from tropical, subtropical, and temperate regions. AVTR: *A. vulgaris* from tropical region, AVSTR: -*A. vulgaris* from subtropical region, and AVT: *A. vulgaris* from temperate region.

Table 3 and Figure 1 provoke the after-effect of anti-inflammatory property, by orally given ethyl acetate and ethanolic fractions of *A. vulgaris* at three different altitudes, in comparison to standard drug Indomethacin, from formalin-induced mouse paw edema. Injection of formalin into mice induced progressive paw edema achieving a maximum at 72 h. In the case of normal control group I, the thickness of the paw was found to be almost unchanged up to 72 h, whereas formalin control group II exhibited an increase in the thickness of the paw every hour and was significant at *p* < 0.05. In all groups treated with standard drug and plant extract, a successive decrease in paw thickness was observed immediately after the oral administration. The decrease in edema was proportional to the time increased. The anti-inflammatory activity of the ethanolic extract was found to be more effective than ethyl acetate extract from all plant samples at different altitudes in a dose-dependent manner. As shown in Figure 2, the highest activity was revealed by the ethanolic extract of *A. vulgaris* from the temperate region. From the time-course curve (at 48 and 72 h), AVTEAE treatment (400 mg/kg) displayed a remarkable effect (*p* < 0.05) with maximal suppressive activity on inflammation by 54.05% at 48 and 72 h, which is almost similar to the standard drug Indomethacin. Besides, the administration of ethyl acetate fraction (400 mg/kg) at the same altitude exhibited moderate effect (*p* < 0.05) with maximal suppressive activity on inflammation by 48.64% at 48 and 72 h. Indomethacin (5 mg/kg) performed the most significant and maximal anti-inflammatory effect by 56.76% at 48 and 72 h. Similarly, the ethanolic leaf extracts from the subtropical region (AVSTREE) and tropical region (AVTEE) displayed paw edema inhibition by 51.35% and 45.94%, respectively, at 48 and 72 h. The comparison of the anti-inflammatory response of *A. vulgaris* ethanolic and ethyl acetate extract, taken from three different altitudes, is illustrated in Figure 2 by plotting a bar diagram where all the extract are given at the dose of 400 mg/kg.

## 4. Discussion

Paw edema, induced in mice by administration of formalin, gives a close resemblance to human arthritis. Hence, it is considered as one of the most suitable techniques to screen possible drugs for chronic inflammation [16]. Formalin induces initial acute inflammatory infiltration into the temporomandibular joint (TMJ) of mice, which later develops into chronic infiltration. Furthermore, it results in persistent cellular proliferation and hyperplasia of the synovial lining and ultimately leads to the formation of villous areas [19]. The mechanism of formalin to induce inflammation is biphasic. In the first phase, its direct effect induces neurogenic pain whereas the second phase is associated with prominent inflammatory reactions governed by serotonin, bradykinin, prostaglandins, histamine, and cytokines [2]. Also, the substantial effect of the hormonal effect in formalin-induced paw edema has not been reported [5, 16], which allowed us to use the Swiss albino mice of either sex in this study. In this experiment, the formalin-induced paw edema model was employed to explore and compare the anti-inflammatory effect *of A. vulgaris* leaf at different altitudes. As shown in Table 3 and Figure 1, the administration of both ethyl acetate and ethanolic extract inhibited the progress of formalin-induced paw edema, depending upon the dose, showing the maximum effect at 48 and 72 h. In previous research, the therapeutic effect of *A. vulgaris* leaf against the acute and subacute inflammatory process has been proved by using carrageenan and cotton pellet granuloma induced inflammatory rat model, respectively [5, 7]. However, the study of its effect against chronic inflammation has not been done yet. The methanolic extract of aerial parts of *A. vulgaris* was found to inhibit carrageenan-induced rat paw edema by 74% at the concentration of 800 mg/kg after 3 h [7]. Similarly, methanolic leaf extract at the concentration of 400 mg/kg had exhibited 55.3% and 66.06% inhibition in the weight of wet and dry cotton pellets, respectively [7]. According to a previous report, *A. vulgaris* extract increases the activity of paraoxonase-1 enzyme. Paraoxonase-1 is a lipophilic antioxidant component, which prevents lipid peroxidation and its serum concentration is decreased during inflammation. Furthermore, it also reduces the increased level of serum TNF-*α* [20]. Diverse classes of bioactive compounds such as flavonoids, sesquiterpenoids, essential oils, tannins, phenols, and saponins present in *A. vulgaris* may produce an anti-inflammatory response by inhibiting the activity of prostaglandins synthesizing enzyme [7]. It has been verified that flavonoids can suppress the pronouncement of inducible nitric oxide synthase isomers, lipoxygenase, and cyclooxygenase, which are key enzymes, involved in the formation of the various inflammatory mediators [21]. Besides that, they can competitively bind to the catalytic site of the ATP to prevent the activity of the regulatory enzyme protein kinase and diminish the inflammatory response [22]. Saponins can exhibit their anti-inflammatory effects by preventing the biological effect of bradykinin or other inflammation mediators, along with the alteration of the prostaglandin synthesis pathway [23]. Similarly, terpenoids can inhibit inflammatory response by impeding the activity of tumor necrosis factor- (TNF-) *α*, cyclooxygenase enzymes, prostaglandin synthesis, cytokines (IL-2, IL-4, and IL-6), and inducible nitric oxide synthase enzymes [24]. Furthermore, the anti-inflammatory potency of tannins and sesquiterpenoids, which are abundantly present in *A. vulgaris*, is associated with the inhibitory effect on cyclooxygenase (COX-2) enzyme [25, 26]. Moreover, further advanced study at the molecular level is needed to establish the exact mechanism of anti-inflammatory action.

Major bioactive compounds present in methanolic and ethanolic extract of *A. vulgaris* are different derivatives of chlorogenic acid (dicaffeoylquinic acid isomers), flavonoids (derivatives of quercetin, rutin, and kaempferol), protocatechuic acid, malic acid, quinic acid, lignans, terpenoids (artemisinic acid glucoside derivatives) [27, 28], and so forth. The proportion of these bioactive compounds extracted by ethanol might be comparatively high as compared to ethyl acetate, due to higher polarity of ethanol than ethyl acetate [29]. This might be the possible reason for the higher anti-inflammatory effect of *A. vulgaris* ethanolic extract in comparison to its ethyl acetate extract.

No significant research work has been conducted, regarding the variation in phytochemical concentration and the pharmacological effect of *A. vulgaris*, growing among different altitudes. In our study, the lowest activity was reported from the plant of lower altitude and it increases with increasing altitude. Various factors such as genetic, phase of growth, soil condition, organogenesis, and anatomical parts may influence the quantitative composition of phytochemicals in medicinal plants and hence their pharmacological activities [30, 31]. Differences in weather conditions and the nature of the soil from which plants are collected (such as altitude variation) and also bring about the difference in phytochemical composition and concentration [30–33]. Essential oils are major bioactive components present in all the species of the genus *Artemisia*. The quantity and quality of essential oils present in *Artemisia* species are always influenced by altitudes and geographical conditions [31]. According to Abad et al., 2012, in the plant *Artemisia nilagirica* grown at different altitudes of Himachal Pradesh India, the concentration of major essential oils present in it showed significant variation with change in altitudes. The concentration of camphor, borneol, and caryophyllene was maximal in higher, middle, and lower altitudes, respectively [34]. Potent anti-inflammatory compound camphor [35, 36] is present abundantly in *A. vulgaris* also [37]. The mechanism for the anti-inflammatory effect of camphor is due to its inhibitory action in the production of interleukin-4, interleukin-2, and TNF-*α* [38]. It may potentially suppress the production of proinflammatory cytokines induced by chemotactic agents like formyl-methionine-leucine-phenylalanine (fMLP), which activate multiple signaling cascades of inflammation [35, 39]. In a previous study, the essential oil content of *Artemisia herba-alba* was studied from four different altitudes; the maximum amount was reported from the plant sample taken at the highest altitudes [40]. In another study of the essential oil from *Artemisia nilagirica*, plants taken at 500 m altitude revealed *α*-thujone as a major compound, but it was present in a very small amount in the plants collected at higher altitudes. In contrast, L-linalool was a major component of the plants taken at 2000 m altitudes whereas it was detected in very low concentration in the case of low altitude plants [41]. L-Linalool is also present in *A. vulgaris* [36] and it is a potent anti-inflammatory compound. It is a competitive antagonist of N-methyl-D-aspartate (NMDA) receptor, which can attenuate behavioral hyperalgesia in rats and it may also prevent the possible action of glutamate release in response to harmful somatic stimuli [42]. For *Artemisia absinthium*, the cytotoxic effect of different plant parts at higher altitudes was found to be increased by 20%–30% in comparison to a lower altitude. The total amount of plant constituents varied from one altitude to another, and the pharmacological activities of such plants would change accordingly [43]. From all these facts, we can conclude that, in *A. vulgaris*, the concentration of anti-inflammatory compounds increases with altitudes which ultimately result in higher activity as compared to lower altitudes.

## 5. Conclusion

In this study, the potency of *A. vulgaris* leaves against chronic inflammation was justified using a valid animal experimental model. Moreover, plants grown at different altitudes exhibit pharmacological effects to a different extent, maybe due to their phytochemical variation. Easy availability and significant potency of *A. vulgaris* with minimum side effects can make this plant an appropriate candidate for the development of novel anti-inflammatory drugs. However, extensive investigation needed to be performed to confirm the effect of the extract via a preclinical and clinical trial with the elucidation of the exact mechanism at the molecular level.

## Figures and Tables

**Table 1:** Information on plant parts, collection area, date, local name, and scientific name.

Collection time	Plant name	Local name	Parts used	Collection site
June 2018	*A. vulgaris*	Tite pati	Leaves	Rupandehi, Arghakhanchi, and Jumla

**Table 2:** Extractive yield of *A. vulgaris* leaves in ethanol and ethyl acetate.

Serial number	Plants	Location	Extractive yield (%)	
			Ethanol extract (%)	Ethyl acetate extract (%)
1	*A. vulgaris*	Tamnar, Rupandehi	6.64	9.21
2	*A. vulgaris*	Dhanchaur, Arghakhanchi	6.62	8.17
3	*A. vulgaris*	Khalanga, Jumla	6.51	8.13

**Table 3:** Effect of ethyl acetate and ethanolic extract of *A. vulgaris* leaves at different altitudes, at the doses of 200 and 400 mg/kg, and indomethacin in comparison to the formalin control group in the formalin-induced paw edema model using a Vernier caliper.

Groups	Dose (ml/kg) p.o.	Change in paw thickness (mm) ± SD (% inhibition)						
		0 hours	1 hour	2 hours	3 hours	24 hours	48 hours	72 hours
I-normal control	500 *µ*L	1.5 ± 0.01	1.5 ± 0.02	1.5 ± 0.02	1.5 ± 0.01	1.5 ± 0.01	1.6 ± 0.02	1.6 ± 0.02

II-formalin control	2 ml/kg	2.4 ± 0.03	4.6 ± 0.05	4.6 ± 0.05,	4.6 ± 0.05,	4.0 ± 0.04	3.7 ± 0.04	3.7 ± 0.04

III-indomethacin	5 mg/kg	1.7 ± 0.04 29.16 ± 1.6%	3.0 ± 0.03 34.78 ± 0.6%	2.7 ± 0.08 41.30 ± 1.7%	2.6 ± 0.03 43.48 ± 0.6%	2.1 ± 0.05 47.50 ± 1.2%	1.6 ± 0.05 56.75 ± 1.3%	1.6 ± 0.06 56.75 ± 1.6%

IV-AVTREE	200 mg/kg	2.2 ± 0.05 8.33 ± 2.0%	4.3 ± 0.07 6.52 ± 1.5%	3.7 ± 0.05 19.56 ± 1.0%	3.2 ± 0.03 30.43 ± 0.6%	2.7 ± 0.06 32.50 ± 1.5%	2.2 ± 0.05 40.54 ± 1.3%	2.2 ± 0.07 40.54 ± 1.9%

V-AVTREE	400 mg/kg	2.0 ± 0.07 16.66 ± 2.9%	3.54 ± 0.04 23.04 ± 0.8%	3.2 ± 0.09 30.43 ± 1.9%	3.0 ± 0.06 34.78 ± 1.3%	2.5 ± 0.08 37.50 ± 2.0%	2.0 ± 0.05 45.94 ± 1.3%	2.0 ± 0.09 45.94 ± 2.4%

VI-AVTREAE	200 mg/kg	2.4 ± 0.08 0 ± 0.8%	4.5 ± 0.05 2.17 ± 1.0%	4.4 ± 0.06 4.35 ± 1.3%	3.4 ± 0.02 26.08 ± 0.4%	3.0 ± 0.07 25.0 ± 1.7%	2.5 ± 0.07 32.43 ± 1.9%	2.3 ± 0.08 37.83 ± 2.1%

VII-AVTREAE	400 mg/kg	2.2 ± 0.05 8.33 ± 2.0%	4.3 ± 0.02 6.52 ± 0.4%	3.5 ± 0.07 23.91 ± 1.5%	3.2 ± 0.06 30.43 ± 1.3%	2.8 ± 0.06 30.0 ± 1.5%	2.2 ± 0.06 40.54 ± 1.6%	2.2 ± 0.05 40.54 ± 1.3%

VIII-AVSTREE	200 mg/kg	2.0 ± 0.01 16.66 ± 0.4%	3.7 ± 0.03 19.56 ± 0.6%	3.2 ± 0.03 30.43 ± 0.6%	3.0 ± 0.07 34.78 ± 1.5%	2.5 ± 0.05 37.5 ± 1.2%	2.0 ± 0.07 45.94 ± 1.9%	2.0 ± 0.05 45.94 ± 1.3%

IX-AVSTREE	400 mg/kg	1.8 ± 0.02 25.0 ± 0.8%	3.3 ± 0.05 28.26 ± 1.0%	3.0 ± 0.03 34.78 ± 0.6%	2.8 ± 0.06 39.13 ± 1.3%	2.3 ± 0.04 42.5 ± 1.0%	1.8 ± 0.07 51.35 ± 1.9%	1.8 ± 0.03 51.35 ± 0.8%

X-AVSTREAE	200 mg/kg	2.0 ± 0.05 16.66 ± 2.0%	4.0 ± 0.07 13.04 ± 1.5%	3.5 ± 0.07 23.91 ± 1.5%	3.2 ± 0.03 30.43 ± 0.6%	2.7 ± 0.06 32.5 ± 1.5%	2.5 ± 0.08 32.43 ± 2.2%	2.1 ± 0.05 43.24 ± 1.3%

XI-AVSTREAE	400 mg/kg	1.8 ± 0.03 25.0 ± 1.2%	3.7 ± 0.04 19.56 ± 0.9%	3.2 ± 0.03 30.43 ± 0.6%	3.0 ± 0.03 34.78 ± 0.6%	2.5 ± 0.04 37.5 ± 1.00%	2.3 ± 0.09 37.84 ± 2.4%	2.0 ± 0.08 45.94 ± 2.2%

XII-AVTEE	200 mg/kg	1.9 ± 0.01 20.83 ± 0.4%	3.6 ± 0.07 21.74 ± 1.5%	3.2 ± 0.01 30.43 ± 0.2%	3.0 ± 0.02 34.78 ± 0.4%	2.5 ± 0.06 37.5 ± 1.5%	1.9 ± 0.06 48.64 ± 1.6%	1.9 ± 0.03 48.64 ± 0.8%

XIII-AVTEE	400 mg/kg	1.7 ± 0.06 29.16 ± 2.5%	3.2 ± 0.06, 30.43 ± 1.3%	2.9 ± 0.03 36.95 ± 0.6%	2.8 ± 0.04 39.13 ± 1.3%	2.2 ± 0.09 45.0 ± 2.2%	1.7 ± 0.05 54.05 ± 1.3%	1.7 ± 0.04 54.05 ± 1.1%

XIV-AVTEAE	200 mg/kg	2.0 ± 0.01 16.66 ± 0.4%	3.9 ± 0.02 15.22 ± 0.4%	3.4 ± 0.05 26.08 ± 1.0%	3.0 ± 0.03 34.78 ± 0.6%	2.6 ± 0.04 35.0 ± 1.0%	2.4 ± 0.04 35.13 ± 1.1%	2.0 ± 0.03 45.94 ± 0.8%

XV-AVTEAE	400 mg/kg	1.8 ± 0.02 25.0 ± 0.8%	3.5 ± 0.01 23.91 ± 0.2%	3.2 ± 0.02 30.43 ± 0.4%	2.9 ± 0.02 36.95 ± 0.4%	2.5 ± 0.03 37.5 ± 1.2%	2.1 ± 0.04 43.24 ± 1.1%	1.9 ± 0.04 48.64 ± 1.1%

All the results are expressed as mean ± SEM; *n* = 5. All the values are statistically significant at *p* < 0.05 in comparison to formalin. AVTREE: *A. vulgaris* tropical ethanolic extract, AVTREAE: *A. vulgaris* tropical ethyl acetate extract, AVSTREE: *A. vulgaris* subtropical ethanolic extract, AVSTREAE: *A. vulgaris* subtropical ethyl acetate extract, AVTEE: *A. vulgaris* temperate ethanolic extract, and AVTEAE: *A. vulgaris* temperate ethyl acetate extract.

## Data Availability

All the data used to support the result of this research are available from Ravin Bhandari upon request.
